# Low-dose xenogeneic mesenchymal stem cells target canine osteoarthritis through systemic immunomodulation and homing

**DOI:** 10.1186/s13075-023-03168-7

**Published:** 2023-10-03

**Authors:** Charlotte Beerts, Sarah Y. Broeckx, Eva Depuydt, Liesa Tack, Lore Van Hecke, Koen Chiers, Leen Van Brantegem, Gabriele Braun, Klaus Hellmann, Nathalie de Bouvre, Nathalie Van Bruaene, Tine De Ryck, Luc Duchateau, Bernadette Van Ryssen, Kathelijne Peremans, Jimmy H. Saunders, Geert Verhoeven, Glenn Pauwelyn, Jan H. Spaas

**Affiliations:** 1 Boehringer Ingelheim Veterinary Medicine Belgium, Noorwegenstraat 4, 9940 Evergem, Belgium; 2 Department of Morphology, Imaging, Orthopedics, Rehabilitation and Nutrition of Domestic Animals, Faculty of Veterinary Medicine, Ghent University, Salisburylaan 133, 9820 Merelbeke, Belgium; 3 Department of Surgery and Anesthesiology of Domestic Animals, Faculty of Veterinary Medicine, Ghent University, Salisburylaan 133, 9820 Merelbeke, Belgium; 4 Department of Pathology, Bacteriology and Poultry diseases, Faculty of Veterinary Medicine, Ghent University, Salisburylaan 133, 9820 Merelbeke, Belgium; 5 Klifovet AG, Geyerspergerstrasse 27, 80689 Munich, Germany; 6 Private Referral Veterinary Practice ‘De Molenkreek’, Polderdreef 31, 4554 AD Westdrope, The Netherlands; 7 Anacura, Noorwegenstraat 4, 9940 Evergem, Belgium; 8 Biometrics Research Group, Faculty of Veterinary Medicine, Ghent University, Salisburylaan 133, 9820 Merelbeke, Belgium; 9 Boehringer Ingelheim Animal Health, 1730 Olympic Drive, Athens, GA 30606 USA

**Keywords:** Mesenchymal stem cells, Xenogeneic, Equine peripheral blood, Immunomodulation, Homing, Osteoarthritis, Canine

## Abstract

**Background:**

As current therapies for canine osteoarthritis (OA) provide mainly symptomatic improvement and fail to address the complex pathology of the disease, mesenchymal stem cells (MSCs) offer a promising biological approach to address both aspects of OA through their immunomodulatory properties.

**Methods:**

This study aimed to investigate the safety and efficacy of xenogeneic MSCs in dogs with OA at different dose levels after intravenous injection. OA was surgically induced in the right stifle joint. Thirty-two male and female dogs were divided into three treatment groups and a control group. Regular general physical examinations; lameness, joint, radiographic, and animal caretaker assessments; pressure plate analyses; and blood analyses were performed over 42 days. At study end, joint tissues were evaluated regarding gross pathology, histopathology, and immunohistochemistry. In a follow-up study, the biodistribution of intravenously injected ^99m^Tc-labeled equine peripheral blood-derived MSCs was evaluated over 24h in three dogs after the cruciate ligament section.

**Results:**

The dose determination study showed the systemic administration of ePB-MSCs in a canine OA model resulted in an analgesic, anti-inflammatory, and joint tissue protective effect associated with improved clinical signs and improved cartilage structure, as well as a good safety profile. Furthermore, a clear dose effect was found with 0.3 × 10^6^ ePB-MSCs as the most effective dose. In addition, this treatment was demonstrated to home specifically towards the injury zone in a biodistribution study.

**Conclusion:**

This model-based study is the first to confirm the efficacy and safety of systemically administered xenogeneic MSCs in dogs with OA. The systemic administration of a low dose of xenogeneic MSCs could offer a widely accessible, safe, and efficacious treatment to address the complex pathology of canine OA and potentially slow down the disease progression by its joint tissue protective effect.

**Supplementary Information:**

The online version contains supplementary material available at 10.1186/s13075-023-03168-7.

## Background

Osteoarthritis (OA) is a progressive inflammatory and degenerative joint disease, causing pain and impaired function in affected joints. It is characterized by the degradation of cartilage tissue causing a chronic low-grade inflammation, osteophyte formation, and subchondral bone remodeling [1]. The most commonly affected joints in dogs are the knee, the hip, the shoulder, and the elbow [2]. Previous prevalence data suggested 20% of dogs over 1 year are affected by OA, whereas new studies support a prevalence of 38% in dogs over 1 year by proactively screening patients for OA signs [2–5].

Currently, canine OA is primarily managed with a multimodal treatment comprising non-steroidal anti-inflammatory drugs (NSAIDs), analgesics, nutraceuticals, exercise management, physiotherapy, and weight control. Although these therapies provide symptomatic improvement and in some cases reduce inflammatory changes, they do not delay the disease progression substantially [6]. Therefore, interest has grown in the use of biological therapies such as mesenchymal stem cells (MSCs) as an alternative treatment to not only control the clinical signs but also address the degenerative nature of OA [7, 8].

The immunomodulatory properties of MSCs have been widely described as they have been shown to switch pro-inflammatory to anti-inflammatory immune reactions, as well as the potential for regeneration by stimulating local repair through paracrine signaling. MSC therapy could provide a long-acting solution to treat canine OA with a potential slowdown of OA progression and maybe even a reversion of the cartilage damage [9–11]. Preclinical studies using different OA models in various species and clinical phase I and II studies, focusing on the intra-articular (IA) administration of autologous MSCs of various tissue sources, have shown promising results for safety and efficacy [12–14]. Other studies also reported the safety and efficacy of IA and intravenous (IV) administered allogeneic MSCs in the treatment of OA. Allogeneic MSCs are clinically more useful than autologous MSCs because they provide a “ready-to-treat” product, avoiding the harvest and production of MSCs for each individual [15–17]. However, the systemic administration of MSCs would make their use even more accessible and might create a more practical solution for patients with OA in multiple joints. The systemic use of MSCs may provide a stronger interaction with the immune system, leading to systemic immunomodulatory and anti-inflammatory effects [17, 18]. Next to autologous and allogeneic MSC treatments, the IA administration of xenogeneic equine peripheral blood-derived MSCs was recently investigated in canine OA patients and shown to be well tolerated and effective in the reduction of lameness and pain [5, 19]. A single intra-articular injection of xenogeneic porcine MSCs in canine stifle joints proved to be safe and effective in the treatment of canine OA [20]. Moreover, a study evaluating the biodistribution pattern of MSCs reported no safety concerns after intravenous, intramuscular, and subcutaneous injection of ^99m^Tc labeled equine peripheral blood-derived MSCs in dogs [21]. Other studies report the intravenous administration of xenogeneic MSCs in swine, mice, and rats [22–27]. The administration of xenogeneic MSCs provides an advantage over allogeneic treatments due to the potential selection of a high-quality donor species, the ability for minimally invasive collection of cells in larger animals, and the absence of transmissible species-specific pathogens [11]. From a medical and economic standpoint, the use of equine xenogeneic MSCs for example is an interesting alternative to allogeneic or autologous MSCs since harvesting tissue from healthy donor horses provides an effective technique to produce highly qualitative MSCs. Finally, xenogeneic MSCs are an interesting alternative for use in dogs since canine MSCs have culture and upscaling limitations caused by senescence earlier in the culture process than for example human and equine MSCs [28–30].

To evaluate the safety and efficacy of xenogeneic MSCs for the treatment of canine OA, a relevant OA model would be needed. Different induced models for OA exist, and the knee is the most used joint in these models. An advantage of surgical models is that the results are highly reproducible and progress quickly. A downside is the difficulty to generate early stages of OA and evaluate an early drug treatment because of the rapid disease induction [31]. The cranial or anterior cruciate ligament transection (ACLT) model is most frequently used to induce OA [2]. However, the transection of the cranial cruciate ligament causes irreversible joint instability, reducing its clinical relevance for naturally occurring osteoarthritis [32]. Another well-described model is the groove model, in which grooves are surgically made in the articular cartilage in weight-bearing regions. In this model, the observed histological and biochemical changes develop over 20 to 40 weeks [32–35]. Finally, in the meniscectomy model, OA is induced by surgical transection of the medial meniscus. In this model, the joint destabilization leads to quick and more severe degeneration than in the ACTL model [36]. The current dose determination study used a combination of abovementioned surgical models to elicit a faster onset of osteoarthritis that will result in a shorter study duration.

To the authors’ knowledge, randomized, controlled, and blinded studies investigating the efficacy and safety of IV-injected xenogeneic MSCs have not been described in any species. In addition, many questions remain about their dosage, mechanisms of action, and potential migration (i.e., homing behavior) in OA patients to the site of the lesion.

Consequently, the potential of systemically administered xenogeneic MSCs in the treatment of canine OA was investigated by evaluating their efficacy and safety at three dose levels in a standardized, controlled canine OA model study. Orthopedic, clinical, hematological, biochemical, pathological, and histopathological parameters were evaluated for safety and efficacy. Furthermore, cellular and humoral immunogenicity as well as immunomodulatory properties of the MSCs were investigated both in vitro and in vivo. In addition, cartilage markers were analyzed in the serum of the dogs to better define the mode of action. In a follow-up biodistribution study, the homing properties of the xenogeneic MSCs to the affected joint were examined using the anterior cruciate ligament transection model.

## Methods

### Study design

This study was a single-center, placebo-controlled, randomized, blinded, and blocked clinical study under laboratory conditions evaluating the efficacy and safety of three different doses of equine peripheral blood-derived mesenchymal stem cells (ePB-MSCs) in an OA model in dogs. The study was blinded by using separate persons for examinations (investigator/examining veterinarian, animal caretaker, and pathologist) and for administration of treatments (dispenser).

In a biodistribution study, the pattern of intravenously administrated ePB-MSCs was evaluated in three dogs following the cruciate ligament section. The experimental unit was the individual animal.

### Sample size and experimental animals

For the dose-response study, 32 healthy purpose-bred Beagles (sixteen males and sixteen females; age: 16 to 19 months; weight: 7.3 to 14.6 kg) were included in the efficacy and safety study and equally divided across four groups based on age and weight. Three healthy purpose-bred Beagles (one male and two females; age: 22 to 23 months) were included in the biodistribution study.

### Housing and husbandry

The dogs were kept in 16 m^2^ pens of 4 dogs each. Feed and water were provided ad libitum.

### Inclusion and exclusion criteria

The included dogs needed to be free of lameness, joint pain, swelling, or heat. Dogs were excluded when showing signs of other diseases prior to the enrollment, unless their joints were authorized by the investigator, the monitor, and the study management. Dogs were excluded if they received non-permitted treatments (NSAIDs or corticosteroids) within 2 months prior to inclusion on day − 21 or MSC therapies. Also, pregnant dogs, lactating dogs, or dogs intended for breeding during the study period were excluded. No dogs were excluded from the study.

### Randomization

During the dose-response study, four animals of the same sex were allocated to each of the eight pens upon arrival. By random catch, the first four dogs of the same sex were allocated to animal IDs 401 to 404 by the examining veterinarian and placed in pen 1, and the second four dogs, again of one sex, were allocated to animal IDs 405 to 408 and placed in pen 2. All further animals were allocated to animal IDs and pens as described above. Animal allocation to pens was recorded on the housing plan. In case animals needed to swap pens, this could be done within animals of the same sex until day − 15, so that the animal batches for surgery were the same as for the randomization on day 0.

### Fate of the study animals

All dogs of the efficacy and safety study were euthanized on day 42 ± 4 for necropsy. The dogs were euthanized by administration of medetomidine (100 μg/kg IM), ketamine (4–8 mg/kg IV), and sodium pentobarbital 20% (0.5–1 mL/kg intracardial). The three dogs of the biodistribution study were housed together in a separate pen and made available for adoption afterwards.

### Blinding

The study was blinded by personnel by using separate people for clinical examinations (examining vet, animal caretaker, pathologist) and for administration of treatments (dispenser).

### Ethical statement

This efficacy study (approval number EC: 2019_003), the biodistribution study (approval number EC: 2019_007), and the blood collection of the donor horses (approval number: EC_2018_002) were approved by an independent ethics committee approved by the Flemish government (recognition number: LA1700607). In addition, the Klifovet Animal Welfare Body evaluated the efficacy study and its procedures according to Directive 2010/63/EU (Klifovet Reg.-No: 18-08), concluding that the study could be conducted as planned. Both clinical studies were compliant with good clinical practices (VICH GL9), and all animal handlings were conducted according to European national and regional animal welfare regulations (Directive 2001/82/EC as amended, Belgian animal welfare legislation (KB 29/05/2013), Directive 2010/63/EU, and EMEA/CVMP/816/00-Final) and the principle of the 3Rs was applied. An informed consent was obtained for all study participants.

### Experimental procedures

Both clinical studies were performed according to a Good Clinical Practice (VICH GL9) compliant protocol.

### Surgical procedure

#### Dose-response study

Two weeks before the treatment administration (day − 14), OA was surgically induced in the right stifle joint of each dog by complete transection of the cranial cruciate ligament, medial meniscal release, and creation of a bilateral cartilage defect on the weight-bearing surfaces of the femoral condyles. Before surgery, dogs were sedated with a combination of medetomidine (80 μg/kg IV or 100 μg/kg intramuscular (IM)) and methadon (100 μg/kg IM or IV). General anesthesia was induced with IV administration of ketamine (8 mg/kg). The right stifle joint of the dog was clipped and prepared for surgery. Adequate anesthesia was achieved by periarticular administration of lidocaine (20 mg/4.5 kg body weight). Since the procedure was short in time and lidocaine (20 mg/4.5 kg bodyweight) was injected peri-articular for pain relief, sufficient depth of anesthesia could be maintained during the entire procedure. A lateral para-patellar arthrotomy was performed with the dog placed in dorsal recumbency. The weight-bearing areas of the femoral condyles were exposed by the placement of stifle distractor(s). The cranial cruciate ligament was transected sharply without damaging other joint structures. A medial meniscal release was performed by transection of the meniscotibial ligament at the caudal pole of the meniscus. A custom-made template from a bend Steinmann pin was used to create 1-cm^2^ cartilage defects on the medial and lateral femoral condyles through debridement with a hand burr. The arthrotomy was closed with a simple interrupted pattern (using 2-0 polydioxanone suture), followed by the closure of the subcutaneous tissue and skin with a simple continuous pattern (using 3-0 polydioxanone suture). To protect the stifle, a bandage was applied for 13 days (day − 13 to day − 1). The bandage was checked daily and changed when necessary. All dogs received pen rest for 1 week (day − 13 to day − 7) and 3 days of post-operative analgesia (buprenorphine 10–20 μg/kg IM) (day − 13 to day − 11). Furthermore, all dogs received a daily clinical assessment after surgery until the end of the study. Only if needed according to a veterinarian assessment, additional pain medication (i.e., rescue treatment) (buprenorphine 10–20 μg/kg IM) was administered after day − 11.

#### Biodistribution study

Three days after arrival, osteoarthritis was surgically induced in the right stifle joint of each dog by transection of the anterior cruciate ligament (ACL). Dogs were sedated with a combination of medetomidine (80 μg/kg IV or 100 μg/kg IM) and methadon (100 μg/kg IM or IV). General anesthesia was induced with ketamine (8 mg/kg) IV. The right stifle joint of the dogs was clipped and prepared for surgery. Lidocaine (20 mg/4.5 kg body weight) was administered periarticular to achieve adequate analgesia. Dogs are placed in dorsal recumbency, and the stifle joint will be draped for surgery. A mini-arthrothomy was performed through a lateral para-patellar tendon incision, under the level of the patella. The anterior cruciate ligament was sharply transected while taking care not to damage the other joint structures. The arthrotomy was closed with a simple interrupted pattern (using 3-0 polydioxanone suture), followed by closure of the subcutaneous tissue and skin with a simple continuous pattern (using 3-0 polydioxanone suture). A bandage was applied for 4 days (day 3 to day 7), which was checked daily and changed when deemed necessary. All dogs were pen rested and received 3 days of post-operative medication (buprenorphine 10–20 μg/kg IM).

On day 0 and day 7, a power Doppler examination was performed on both stifle joints of each dog. If needed, dogs were sedated with dexmedetomidine. Dogs were placed in lateral and/or ventral recumbency. Both stifle joint areas of the dogs were clipped, and a gel was applied before positioning the ultrasound probe. The vascularization of the stifle joint was evaluated and documented in a report by the imaging expert.

### Isolation and cultivation of MSCs

According to previously described methods, the MSCs were isolated from the venous blood collected from the vena jugularis of two donor horses. As described by Broeckx, the donor horses were tested for the presence of multiple transmittable diseases prior to blood collection [37]. The cells were cultivated in a Good Manufacturing Practice (GMP)-certified production site (BE/GMP/2018/123) according to GMP guidelines until passage (P) 5 and characterized on viability, morphology, presence of cell surface markers, population doubling time (acceptance criteria are between 0.7 and 3.0), and trilinear differentiation. Evaluation of the presence (cluster of differentiation (CD) 29, CD44, and CD90) and absence (major histocompatibility complex (MHC) II, CD45, and marker for monocytes and macrophages) of specific cell surface markers was accomplished by flow cytometry as previously described [30]. Consequently, the MSCs were cultivated until P10, trypsinized, and resuspended to a final concentration of 0.3 × 10^6^ cells/mL in Dulbecco’s modified Eagle medium (DMEM) low glucose with 10% dimethylsulfoxide (DMSO). The MSCs were stored at − 80 °C in cryovials until further use. The sterility of the final product was confirmed by the absence of aerobic bacteria, anaerobic bacteria, fungi, endotoxins, and mycoplasma. Two batches from two different donor horses were used in the efficacy study. Only one batch was used in the biodistribution study.

### Treatment administration

On the day of treatment (day 0), the thirty-two purpose-bred dogs were randomly allocated to four treatment groups of eight dogs (T1, T2, T3, and T4). The dogs of treatment groups T1, T2, and T3 received an IV injection in the cephalic vein at the level of the mid-third of the radius with respectively 0.06 × 10^6^ MSCs (0.2 mL), 0.3 × 10^6^ MSCs (1 mL), and 1.5 × 10^6^ (5 mL) MSCs. The MSCs were injected immediately after thawing the vial in the palm of the hand. The dogs in treatment group T4, the control group, were intravenously injected with 5 mL 0.9% NaCl solution (Vetivex 9 mg/mL). To ensure that the correct product was administered to each treatment group, the dispenser was not blinded. The dispenser did not take part in any of the assessments. The animal caretaker and the investigator/examining veterinarian were blinded and consequently were never involved in any treatment allocation and neither observed nor were involved in any treatment administration at any time of the study nor had access to such information until the time of unblinding.

### Safety and efficacy outcome measures

#### General physical assessment

A general physical examination of the dogs was conducted daily throughout the study by the same veterinarian. All relevant organ systems, body temperature, respiratory rate, pulse rate, mucous membrane color, and capillary refill time were examined.

#### Lameness and joint assessment

**Fig. 1:**
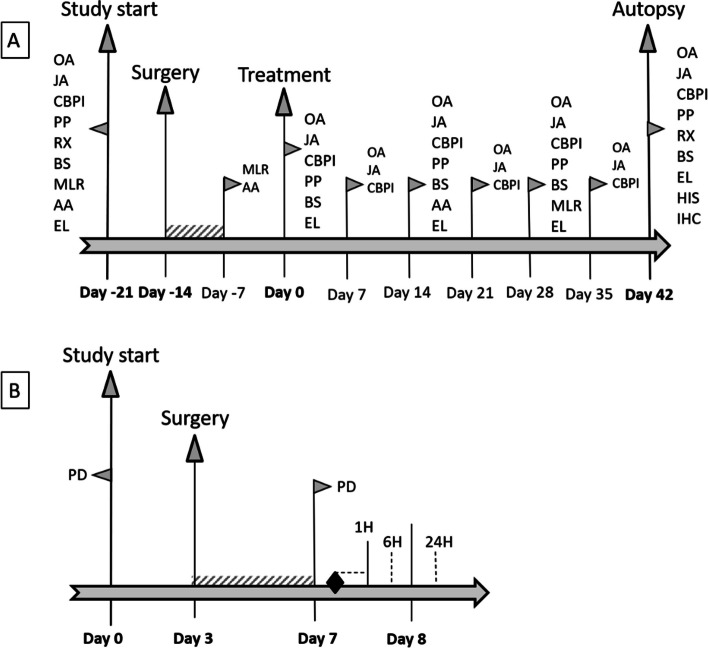
Study overview representing the different study procedures (**A**) and the biodistribution examinations (**B**). “  ” indicates pen rest. “  ” indicates ^99m^Tc-labeled equine peripheral blood-derived mesenchymal stem cell administration. AA, blood sampling for antibody assay; OA, orthopedic assessment; EL, blood sampling for ELISA; JA, joint assessment; HIS, histopathology; CBPI, Canine Brief Pain Inventory; IHC, immunohistochemistry; PP, pressure plate analysis; PD, power Doppler examination; RX, radiographic examination: 6 succesive total body scans; BS, blood sampling: 1 total body scan; MLR, blood sampling for mixed lymphocyte reaction

Lameness, range of motion (ROM), articular heat, joint effusion, and articular pain were assessed prior to treatment on day − 21 and on day 0 by the same veterinarian. Post-treatment assessment was performed weekly until the end of the study (day 42 ± 4) (Fig. 1). Lameness assessment was performed according to a scoring system based on the degree of lameness (score 0 to 4), respectively clinically normal to nearly incapacitated dog, adapted from the scale described by Robinson and Shave and Payne-Johnson et al. [38, 39]. Evaluation of ROM in degrees (°) was assessed by the use of a goniometer [40]. Similar scoring systems were applied to evaluate articular heat (score 0 or 1, respectively absence to presence of heat), joint effusion (score 0 to 3, respectively none to severe signs of effusion; according to Millis and Levine), and articular pain (score 0 to 4, respectively no pain to nearly incapacitated dog; adapted from Robinson and Shave and Payne-Johnson et al. [38, 39]).

#### Radiographic assessment

Radiographic assessment of the stifle joints was performed on day − 21 and on day 42 ± 4 under sedation with dexmedetomidine (25 μg/kg IM or 5 μg/kg IV) according to Wessely et al. [41] (Fig. 1). On the mediolateral and the caudocranial view, 15 pre-defined structures were scored on the presence of radiographic abnormalities (score 1 to 4, none to marked abnormality). The scores of all 15 structures were summed to an overall radiographic score.

#### Animal caretaker assessment

To assess the pain and quality of life of each dog, a Canine Brief Pain Inventory (CBPI) assessment was performed. The CBPI is composed of a pain severity score (PSS) assessing the pain at its worst, least, and average, in the last 7 days, and now, a pain interference score (PIS) to measure how much pain interferes, in the last 7 days, with six daily activities and questions related to quality of life [42]. The CBPI assessment forms were completed by a single caretaker on day − 21, day 0, and then weekly until the end of the study (day 42 ± 4) (Fig. 1).

#### Pressure plate analysis

Objective gait data was obtained by pressure plate analysis (Zebris Medical GmbH) on day − 21, day 0, day 14 ± 1, day 28 ± 1, and day 42 ± 4 (Fig. 1). Before data acquisition (day − 35 (± 4) to day − 22), dogs were allowed a habituation period walking across the walkway. During the analysis, one handler guides the dog across the walkway. One trial is defined as the dog walking the entire length of the walkway in one direction. A trial is considered valid if the dog moved in a straight line without pulling to one side or turning its head at a visually constant pace, the four paws fully contacting the plate surface. The values of each parameter per run will be determined by the designated software. A minimum of five valid trials are collected at each analysis. During the trials, the following parameters are measured: mean force (MF), symmetry index of the mean force of the hind legs (%), and mean maximal force (MMF). The mean value of the right hind leg for each parameter over the first 5 valid runs was calculated and noted for all parameters.

#### Blood sample collection and laboratory analysis

On day 0 (prior to treatment), 14 ± 1, 28 ± 1, and 42 ± 4, blood samples were collected (Fig. 1). At least 2.0 mL blood in ethylenediaminetetraacetic acid (EDTA) tubes was collected for standard hematology panel. For standard biochemical analysis, at least 4 mL of blood was sampled in serum tubes (containing a gel separator to enhance blood clotting), and at least 0.5 mL of blood was sampled on fluoride for glucose measurement.

#### Gross pathology, histopathology, and immunohistochemistry

After sedation and induction of anesthesia, the dogs were euthanized on day 42 ± 4 by administration of medetomidine (80 μg/kg IV or 100 μg/kg IM), ketamine (4–8 mg/kg IV), and sodium pentobarbital 20% (0.5–1 mL/kg IV or intracardial). Post-mortem examinations consisting of gross pathology, histopathology, and immunohistochemistry according to Osteoarthritis Research Society International (OARSI) recommendations were performed as described by Cook et al. under the responsibility of a European College of Veterinary Pathologists certified pathologist [43] (Additional file 1). The pathologist was blinded.

The assessment of gross pathology was based on synovial pathology, cartilage pathology of the weight-bearing surfaces of the medial femoral condyle (MFC) and the lateral femoral condyle (LFC), pathology of the medial and lateral meniscus, and evaluation of ectopic tissue at the injection site. Synovial pathology of the affected joint was scored based on the worst pathology observed (score 0 to 5, respectively normal to severe pathology). Also, cartilage pathology was scored based on the most severe pathology noted (score 0 to 4, respectively smooth cartilage surface to large areas of severe cartilage damage). Pathology of the menisci was assessed separately for each meniscus. Each meniscus was scored (score 0 to 4, respectively no pathology to complete destruction) per area (anterior, middle, posterior) to calculate a total score per meniscus. The skin and blood vessels at the injection site were assessed for the presence of ectopic tissue (score 0 to 1, respectively absent to present).

All histopathology samples were fixed in a 4% formaldehyde solution, embedded in paraffin, sectioned at 4 μm thickness, and stained with hematoxylin and eosin. Histopathology was performed to evaluate the joint surface, synovium, and ectopic tissue at the MFC (adjacent to lesion), the LFC (adjacent to lesion), the medial tibial plateau, the lateral tibial plateau, and the injection site. Histopathologic assessment of the joint surface focused on scoring the cartilage structure and chondrocyte pathology (score 0 to 12, respectively normal to full thickness cartilage loss and cell loss). The synovium was assessed by evaluating the amount of cell infiltration (score 0 to 6, respectively no cell infiltration to marked cell infiltration). Ectopic tissue was scored based on its presence (score 0 to 1, respectively absent to present).

An immunohistochemical assessment of the affected joint was performed at predefined locations adjacent to the cartilage lesions for the different components of the cartilage of the MFC and the LFC joint surfaces to evaluate cartilage oligomeric matrix protein (COMP), collagen type II, Alcian blue stain for glycosaminoglycan, and von Willebrand factor (vWF). Additionally, the vascularity in the synovium/joint capsule was assessed.

### Immunogenicity analysis

#### Flow cytometric crossmatch assay

MSCs from the same batch as used for the treatment of the dogs were thawed and resuspended in HBSS 1× to a final concentration of 2 × 10^5^ cells/mL. One milliliter of the cell suspension was centrifuged and blocked with normal rabbit serum. Next, heat-inactivated serum derived from each dog before (day − 7, baseline) and after treatment (day 28) was co-incubated for 30 min. Subsequently, a secondary rabbit anti-dog IgG antibody labeled with Alexa Fluor 647 (Jackson Immunoresearch) was added and incubated for 20 min at room temperature. After incubation, all the samples were stained with 7-AAD for flow cytometric analysis (BD FACSCanto II, BD Biosciences, USA).

As a positive control, equine peripheral blood mononuclear cells (PBMCs) were co-incubated with normal canine serum to confirm the secondary antibody detects cells bound to the primary antibodies appropriately. The negative control sample consisted of MSCs co-incubated with the secondary antibody without the addition of canine serum. All samples were analyzed in duplicate, and the mean values were calculated and used for statistical comparison**.**

### Immunomodulation analysis

#### Modified mixed lymphocyte reaction (MLR)

The MLR assay serves two purposes in this study: first, the MLR was used to evaluate the cellular immunogenicity towards the MSCs; second, the MLR was used to evaluate the immunomodulatory properties of the MSCs against PBMCs derived from dogs before and after treatment with MSCs.

The MLR assay was performed for all dogs after surgery (day − 7) and after treatment administration on day 28 (Fig. 1). The whole blood was collected from the dogs using sterile K_3_EDTA tubes and layered upon a Percoll density gradient before centrifugation. Next, the interphase fraction containing the canine peripheral blood mononuclear cells (PBMCs) was collected and washed with Hank’s Balanced Salt Solution 1× (HBSS). Consequently, the PBMCs were resuspended in HBSS 1× and labeled with carboxyfluorescein succinimidyl ester (CFSE) according to the manufacturer’s instructions (Life Technologies) to evaluate cell proliferation. Finally, the PBMCs were diluted in a culture medium (DMEM supplemented with FBS, AB/AM, and β-mercapto-ethanol (BME; Sigma)) to a final concentration of 2 × 10^6^ cells/mL. One hundred microliters of the cell suspension was added to the designated wells of a U-bottom 96-well plate. Prior to the PBMC isolation, MSCs were thawed, washed, and resuspended in culture medium to a final concentration of 2 × 10^5^ MSCs/mL. These MSCs were plated at a ratio of 1:10 MSCs:PBMCs 24 h before adding the labeled PBMCs. To assess the immunomodulatory properties of the MSCs towards stimulated PBMCs derived from dogs untreated and treated with MSCs, the cells were co-incubated with concanavalin A (con A) (5 μg/mL, Sigma Aldrich) stimulated PBMCs. Finally, a negative control containing only canine PBMCs was added to the setup for each dog to assess baseline proliferation. As a positive control, PBMCs from each dog were stimulated with the mitogen con A (5 μg/mL, Sigma Aldrich). The 96-well plate was incubated for 4 days in a humidified incubator at 37 °C and 5% CO_2_.

After incubation, all samples were stained for cell viability with 7-aminoactinomycine D (7-AAD; BioLegend, USA). Subsequently, the PBMC proliferation (%) was measured using flow cytometry analysis of the CFSE staining (BD FACSCanto II, BD Biosciences, USA). All samples were analyzed in duplicate, and the mean values were calculated and used for statistical comparison.

An ELISA experiment was performed using commercial ELISA kits (R&D systems) to measure PGE2 concentration in the supernatants of the immunomodulatory samples, according to the manufacturers’ instructions. All samples were analyzed in duplicate, and the mean values were calculated and used for statistical comparison.

#### ELISA

ELISA was performed on the serum of the dogs to gain more insights into the in vivo immunomodulatory properties of the ePB-MSCs in dogs suffering from OA by measuring a well-known immunomodulatory marker released by stem cells (PGE2) and pro-inflammatory cytokines (TGF-β1, C3a, IFN-γ) as well as further potential biomarkers (HA and C2C).

ELISA analysis was performed on the canine serum samples collected on day − 21, day 0, day 14 ± 1, day 28 ± 1, and day 42 ± 4 for prostaglandin E2 (PGE2), interferon-gamma (IFN-γ), transforming growth factor beta 1 (TGF-β1), hyaluronic acid (HA), complement factor C3a (C3a), and collagen type II cleavage (C2C) (Fig. 1). All ELISA experiments were performed using commercial ELISA kits (PGE2 (R&D systems), IFN-γ (Cloud clone corp.), TGF-β1 (Cloud Clone Corp.), HA (TECOmedical), C3a (Cloud Clone Corp.), and C2C (MybioSource) according to the manufacturers’ instructions. All samples were analyzed in duplicate, and the mean values were calculated and used for statistical comparison.

### Biodistribution study

On day 7 after the Doppler examination, the biodistribution of MSCs to injured and healthy stifle joints during 24 h post-injection was examined using ^99m^Technetium (^99m^Tc)-labeled MSCs in three dogs. The radioactivity in both stifle joints (control and lesion) was quantified using manually drawn regions of interest (ROI) on the lateral view of the whole-body scans. Relative uptake of MSCs in the lesion joint was expressed as a fold increase in measured counts over the control lesion. ^99m^Tc labeling was performed as described by Beerts et al. [21]. Briefly, 0.9–1 × 10^6^ MSCs were re-suspended in saline and mixed with SnCl_2_ (Sigma Aldrich, USA) dissolved in sterile basic water (pH 8.5) and 1665 ± 185 MBq of ^99m^Tc (GE Health Care, Eindhoven, The Netherlands). The mixture was incubated for 30 min at room temperature and washed with DMEM. The ^99m^Tc-labeled MSCs, resuspended in saline, were administered through a 22-gauge catheter in one of the cephalic veins at the level of the mid-third of the radius. During the first hour, 6 h, and 24 h post-injection, a scintigraphy examination was performed of the healthy and injured joint using a two-headed gamma camera, equipped with low-energy high-resolution collimators (GCA 7200 A; Toshiba) (Fig. 1). Before acquisition, the dogs were sedated with dexmedetomidine (12–25 μg/kg IM), followed by an anesthesia induced using propofol (dosage on effect) and maintained with isoflurane 1.2–1.4% (on effect) in 100% oxygen after endotracheal intubation. The radioactivity in both stifle joints (control and lesion) was quantified using manually drawn regions of interest (ROI) on the lateral view of the whole-body scans (matrix size 512 × 1024) using the free-hand region of interest tool of a DICOM viewing software platform (Hermes MultiModalityTM, Nuclear Diagnostics, Sweden). Relative uptake of MSCs in the lesion joint was expressed as a fold increase in measured counts over the control lesion.

### Quantification and statistical analysis

#### Efficacy parameters

Changes in lameness, articular pain, CBPI, joint effusion scores, range of motion, and pressure plate analysis (mean force, mean maximum force, and symmetry index) from day 0 to day 42 ± 4 were compared between all IVP groups (T1, T2, and T3) and the control group (T4) using ANOVA. A global significance level of 5% was used, and a comparison-wise significance level was adapted according to Bonferroni correction, i.e., with three comparisons (treatments compared pairwise with control) a comparison-wise significance level of 0.05/3 = 0.0167 was used.

To compare the frequency of synovitis and cartilage scores between all IVP groups and the control group, the Mantel-Haenszel test was used. The frequency of total scores of each meniscus was compared between the treatment groups with the Kruskal-Wallis test. Histopathological and immunohistochemical parameters (COMP, collagen type II, Alcian blue stain for glycosaminoglycan, and vWF) were reported descriptively.

#### Flow cytometric crossmatch assay

Changes in the samples and controls were compared over time within each treatment group using a paired sample *T*-test, and *P*-values below 0.0125 were considered statically significant (Bonferroni correction 0.05/4).

#### MLR

The Friedman test was used to test the difference between immunogenicity and immunomodulation at day − 7, day 0, and day 28. The Kruskal-Wallis 1-way ANOVA test was used to compare the negative control to the immunogenicity samples and the positive control to the immunomodulation samples before and after treatment.

#### ELISA of the serum

The change from baseline for each IVP group (T1, T2, and T3) was statistically compared to the control group T4. When the normal distribution assumption could not be rejected, the analysis was based on the *T*-test, otherwise on the Mann-Whitney *U* test. *P*-values ≤ 0.0167 were considered statistically significant (Bonferroni correction 0.05/3).

#### Correlation between ELISA and clinical findings

Based on the synovial and cartilage gross pathology scores on day 42, dogs were categorized into two groups. In the group labeled as “cases,” all dogs with at least one of both scores > 2 were included. The group labeled as “controls” are the dogs with a maximum of one of the synovial or cartilage scores equal to 2. Distributions of the controls and cases in relation to the ELISA results are evaluated using chi-squared analysis for both categorizations. To evaluate the univariate shifts between the cases and controls at the different time points, *T*-tests or Mann-Whitney *U* tests were performed depending on the normality as evaluated by Shapiro-Wilk.

## Results

### General clinical and physical assessment

No additional pain medication (i.e., rescue treatment) (buprenorphine 10–20 μg/kg IM) was administered after day − 11. During the entire study period, no adverse clinical events or alterations in general physical health were reported. Furthermore, there were no clinically relevant hematological and biochemical changes detected.

### Orthopedic assessment

**Fig. 2:**
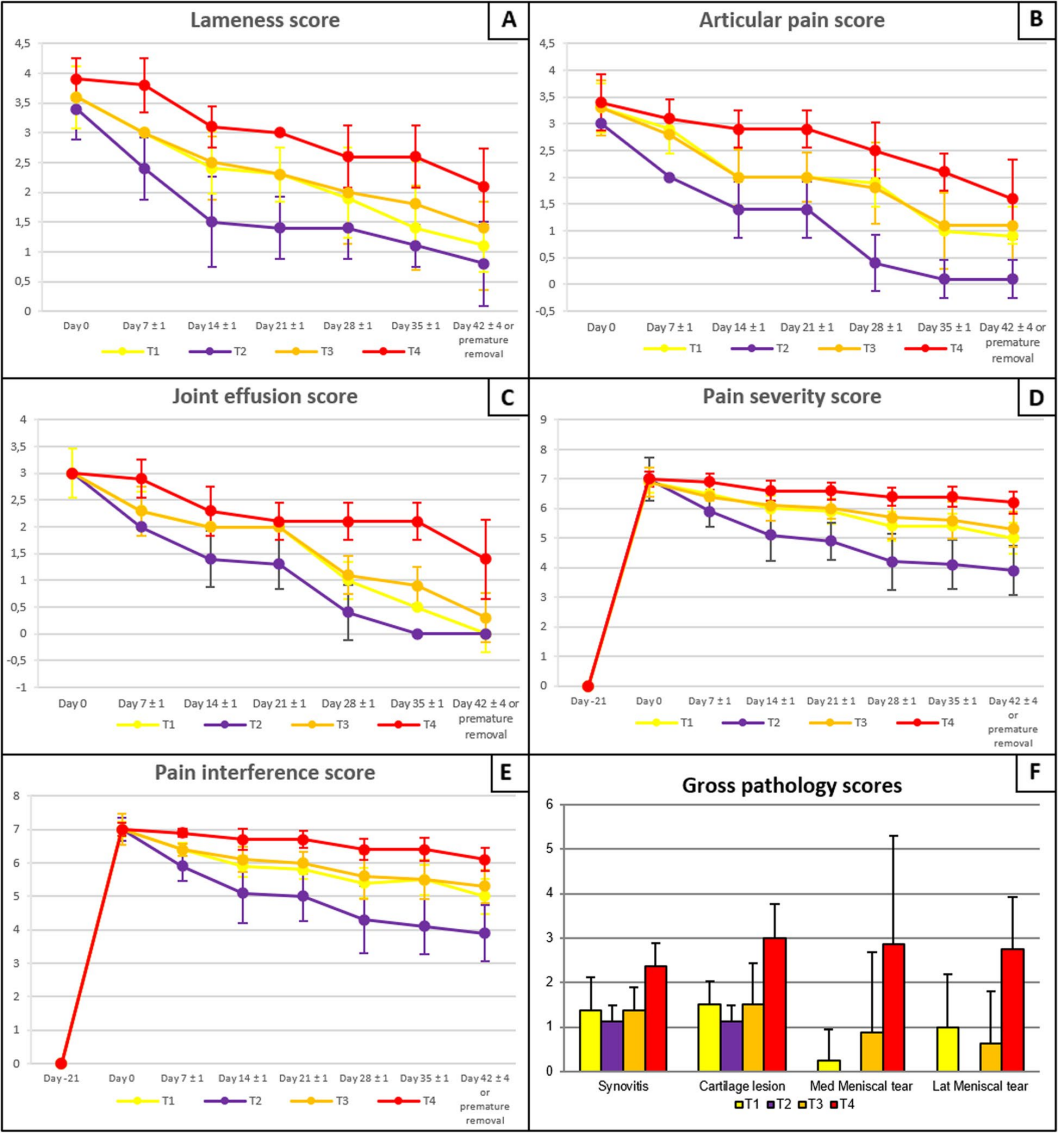
Representation of the orthopedic assessment and CBPI over time and gross pathology results. Treatment groups (± SD) over time are shown for lameness (**A**), articular pain (**B**), joint effusion (**C**), pain severity (**D**), pain interference (**E**), and gross pathology (**F**). Data presented as mean ± SD

**Fig. 3:**
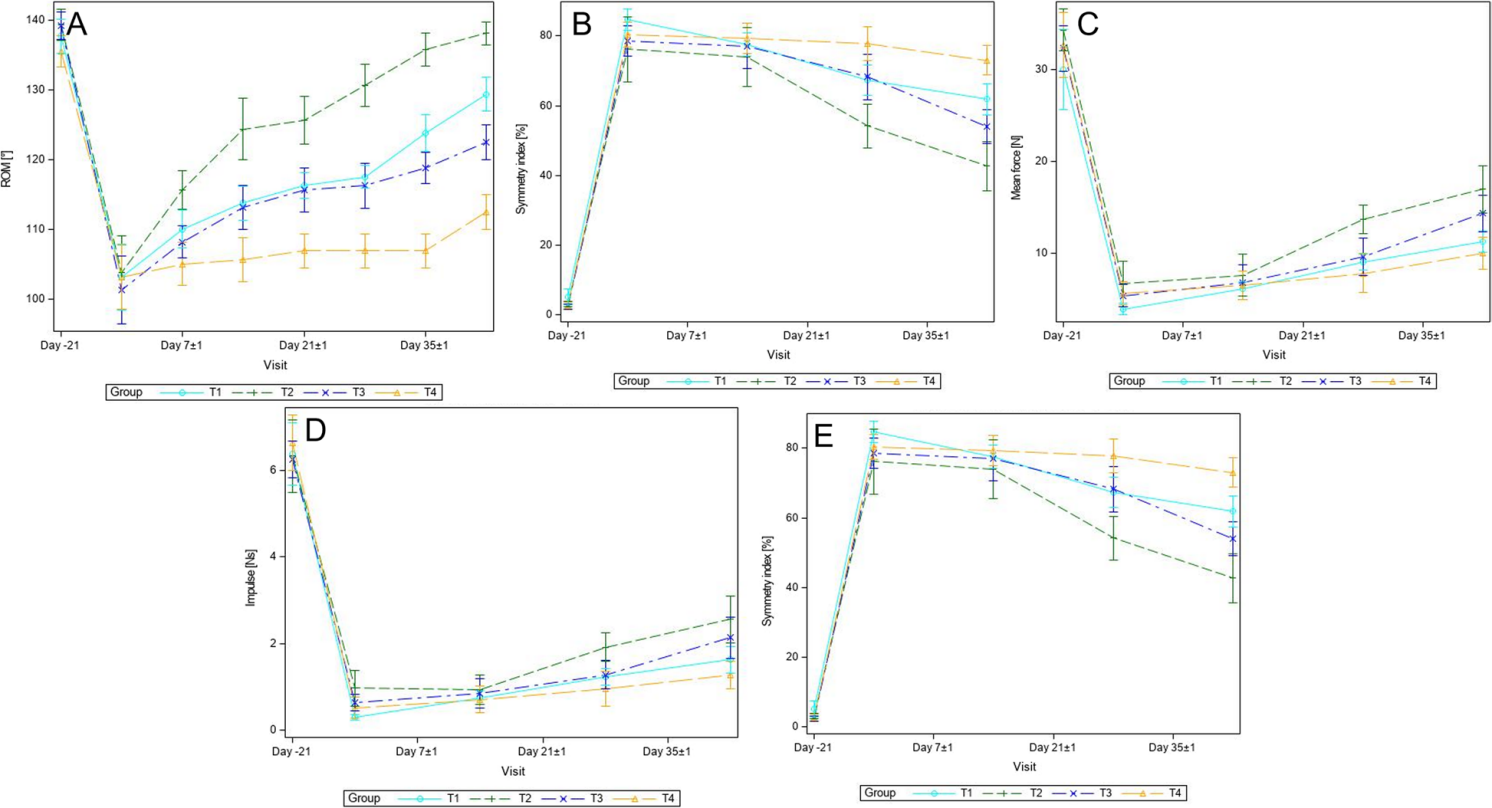
Representation of the assessment of the range of motion and the pressure plate analysis. **A** Range of motion (%) by group over time (mean ± SD). **B** Mean maximum force (*N*) by group over time (mean ± SD). **C** Mean force (*N*) by group over time (mean ± SD). **D** Mean impulse (*N*) by group over time (mean ± SD). **E** Symmetry index (*N*) by group over time (mean ± SD)

At each of the post-baseline visits, lower lameness, articular pain, and joint effusion scores were found in all three investigational veterinary product (IVP) groups (T1: 0.06 × 10^6^ equine peripheral blood-derived mesenchymal stem cells (ePB-MSCs), T2: 0.3 × 10^6^ ePB-MSCs and T3: 1.5 × 10^6^ ePB-MSCs) compared to the control group (T4: 5 mL 0.9% NaCl). At the study end, on day 42 ± 4, lameness scores were significantly lower for group T1 (*P* = 0.0043) and T2 (*P* = 0.0142) compared to T4. Articular pain scores were significantly lower for group T2 (*P* = 0.0015) compared to T4 at day 42 ± 4. Joint effusion scores were significantly lower for all IVP groups (T1: *P* = 0.0001, T2: *P* = 0.0001, T3: *P* = 0.0001) compared to T4 at day 42 ± 4. Generally, the highest score reduction was observed in group T2 (Fig. 2A–C). From day 14 ± 1 onwards, higher range of motion (ROM) values were found in all three IVP groups compared to group T4 with the highest increase for group T2 at all post-baseline visits (Fig. 3A). ROM scores were significantly higher for group T1 (*P* < 0.001), T2 (*P* < 0.001), and T3 (*P* = 0.004) compared to T4 at day 42 ± 4. The pressure plate analysis revealed increases in maximum force (MF), mean maximum force (MMF), and symmetry index (SI) in each of the three IVP groups, with the highest increases in group T2 (Fig. 3B–E). However, a significant pairwise difference was found only for the comparison between the mean force of group T2 (*P* = 0.016) and T4 at day 42 ± 4.

### CBPI assessment

At each of the post-baseline visits, the mean pain severity score (PSS) and pain interference score (PIS) were lower in the three IVP groups compared to T4 and significantly different at day 42 ± 4 (*P* < 0.0001). Group T2 showed the highest score reduction for both PSS and PIS on day 42 ± 4 (Fig. 2D, E). For quality of life (QOL), more animals had a score of 2 (= very good) or 3 (= good) in all three IVP groups compared to the group T4 from day 7 ± 1 onwards. Group T2 showed the highest percentage of animals with score 2 on day 42 ± 4 and was significantly higher than T4 (*P* = 0.0007) (Table 1).

**Table 1:** CBPI: quality of life—frequency of scores

Visit	Score	T1 (*N* = 8), *N* (%)	T2 (*N* = 8), *N* (%)	T3 (*N* = 8), *N* (%)	T4 (*N* = 8), *N* (%)	*P*-value Mantel-Haenszel test
Day − 21	1 = excellent	8 (100.0%)	8 (100.0%)	8 (100.0%)	8 (100.0%)	Not done
Day 0	4 = fair	8 (100.0%)	8 (100.0%)	8 (100.0%)	8 (100.0%)	Not done
Day 7 ± 1	3 = good	2 ( 25.0%)	6 (75.0%)	3 (37.5%)	0 (0.0%)	
	4 = fair	6 (75.0%)	2 (25.0%)	5 (62.5%)	8 (100.0%)	**0.018**
	Total	8 (100.0%)	8 (100.0%)	8 (100.0%)	8 (100.0%)	
Day 14 ± 1	2 = very good	0 (0.0%)	2 (25.0%)	0 (0.0%)	0 (0.0%)	
	3 = good	4 (50.0%)	4 (50.0%)	4 (50.0%)	0 (0.0%)	**0.018**
	4 = fair	4 (50.0%)	2 (25.0%)	4 (50.0%)	8 (100.0%)	
	Total	8 (100.0%)	8 (100.0%)	8 (100.0%)	8 (100.0%)	
Day 21 ± 1	2 = very good	0 (0.0%)	2 (25.0%)	0 (0.0%)	0 (0.0%)	
	3 = good	7 (87.5%)	6 (75.0%)	6 (75.0%)	0 (0.0%)	**< 0.001**
	4 = fair	1 (12.5%)	0 (0.0%)	2 (25.0%)	8 (100.0%)	
	Total	8 (100.0%)	8 (100.0%)	8 (100.0%)	8 (100.0%)	
Day 28 ± 1	2 = very good	0 (0.0%)	5 (62.5%)	0 (0.0%)	0 (0.0%)	
	3 = good	8 (100.0%)	3 (37.5%)	6 (75.0%)	4 (50.0%)	**0.001**
	4 = fair	0 (0.0%)	0 (0.0%)	2 (25.0%)	4 (50.0%)	
	Total	8 (100.0%)	8 (100.0%)	8 (100.0%)	8 (100.0%)	
Day 35 ± 1	2 = very good	0 (0.0%)	5 (62.5%)	0 (0.0%)	0 (0.0%)	
	3 = good	8 (100.0%)	3 (37.5%)	7 (87.5%)	5 (62.5%)	**0.002**
	4 = fair	0 (0.0%)	0 (0.0%)	1 (12.5%)	3 (37.5%)	
	Total	8 (100.0%)	8 (100.0%)	8 (100.0%)	8 (100.0%)	
Day 42 ± 4 or premature removal	2 = very good	1 (12.5%)	6 (75.0%)	0 (0.0%)	0 (0.0%)	
	3 = good	7 (87.5%)	2 (25.0%)	7 (87.5%)	6 (75.0%)	**0.001**
	4 = fair	0 (0.0%)	0 (0.0%)	1 (12.5%)	2 (25.0%)	
	Total	8 (100.0%)	8 (100.0%)	8 (100.0%)	8 (100.0%)	

### Gross pathology, histopathology, and immunohistochemistry assessments at day 42 ± 4

In gross pathology, the synovitis and cartilage scores were significantly lower for all three IVP groups compared to T4 (synovitis score: T1: *P* = 0.010, T2: *P* < 0.001, T3: *P* = 0.005; cartilage score: T1: *P* = 0.002, T2: *P* < 0.001, T3: *P* = 0.008). The highest incidence of score 1 (= normal) for synovitis and cartilage scores was observed in group T2, with only slight to mild changes in all dogs of groups T2 and T3 compared to only 62.5% in T4. The score for both menisci was only significantly lower in the IVP group T2 compared to T4 (lateral: *P* = 0.003; medial: *P* = 0.012) (Fig. 2F; Tables 2, 3, and 4; Additional file 1: Fig. S5).

**Table 2:** Synovial pathology—frequencies of scores

Day 42 ± 4 or premature removal	T1 (*N* = 8), *N* (%)	T2 (*N* = 8), *N* (%)	T3 (*N* = 8), *N* (%)	T4 (*N* = 8), *N* (%)	*P*-value Mantel-Haenszel test	T1 vs. T4	T2 vs. T4	T3 vs. T4
1 = slight	6 (75.0%)	7 (87.5%)	5 (62.5%)	0 (0.0%)				
2 = mild	1 (12.5%)	1 (12.5%)	3 (37.5%)	5 (62.5%)	**0.002**	**0.010**	**< 0.001**	**0.005**
3 = moderate	1 (12.5%)	0 (0.0%)	0 (0.0%)	3 (37.5%)				
Total	8 (100.0%)	8 (100.0%)	8 (100.0%)	8 (100.0%)

**Table 3:** Cartilage pathology of MFC and LFC—frequencies of scores

Day 42 ± 4 or premature removal	T1 (*N* = 8), *N* (%)	T2 (*N* = 8), *N* (%)	T3 (*N* = 8), *N* (%)	T4 (*N* = 8), *N* (%)	Global test	*P*-value Mantel-Haenszel test		
						T1 vs. T4	T2 vs. T4	T3 vs. T4
1 = slightly fibrillated/roughened surface	4 (50.0%)	7 (87.5%)	6 (75.0%)	0 (0.0%)				
2 = fibrillated surface with focal partial thickness lesions	4 (50.0%)	1 (12.5%)	0 (0.0%)	2 (25.0%)	< 0.001	0.002	< 0.001	0.008
3 = deep lesions with surrounding damage	0 (0.0%)	0 (0.0%)	2 (25.0%)	4 (50.0%)				
4 = large areas of severe damage	0 (0.0%)	0 (0.0%)	0 (0.0%)	2 (25.0%)				
Total	8 (100.0%)	8 (100.0%)	8 (100.0%)	8 (100.0%)

**Table 4:** Medial and lateral meniscus pathology—frequencies of scores

Day 42 ± 4 or premature removal	T1 (*N* = 8)	T2 (*N* = 8)	T3 (*N* = 8)	T4 (*N* = 8)	Global test	T1 vs. T4	T2 vs. T4	T3 vs. T4
**Medial meniscus**								
*n*	8	8	8	8				
Mean (SD)	0.3 (0.71)	0.0 (0.00)	0.9 (1.81)	2.9 (2.42)	**0.005**	**0.025**	**0.012**	0.092
Min-max	0–2	0–0	0–5	0–7				
Median	0.0	0.0	0.0	2.5				
Q1–Q3	0.0–0.0	0.0–0.0	0.0–1.0	1.0–4.5				
**Lateral meniscus**								
*n*	8	8	8	8				
Mean (SD)	1.0 (1.20)	0.0 (0.00)	0.6 (1.19)	2.8 (1.16)	**< 0.001**	**0.035**	**0.003**	**0.017**
Min-max	0–3	0–0	0–3	1–4				
Median	0.5	0.0	0.0	2.5				
Q1–Q3	0.0–2.0	0.0–0.0	0.0–1.0	2.0–4.0				

Histopathologic evaluations of the joint surface adjacent to the cartilage defects and the synovium provided similar results for all groups. No significant differences could be found for the immunohistochemistry assessments adjacent to the defects of cartilage oligometric matrix protein (COMP), collagen type II, glycosaminoglycan, and von Willebrand factor (vWF) between the treatment groups (Additional file 1: Supplementary information). Finally, no ectopic tissue was found at the injection site, medial femoral condyle (MFC), lateral femoral condyle (LFC), medial tibial plateau, and lateral tibial plateau.

### Immunogenicity assessment

#### Flow cytometric crossmatch assay

**Fig. 4:**
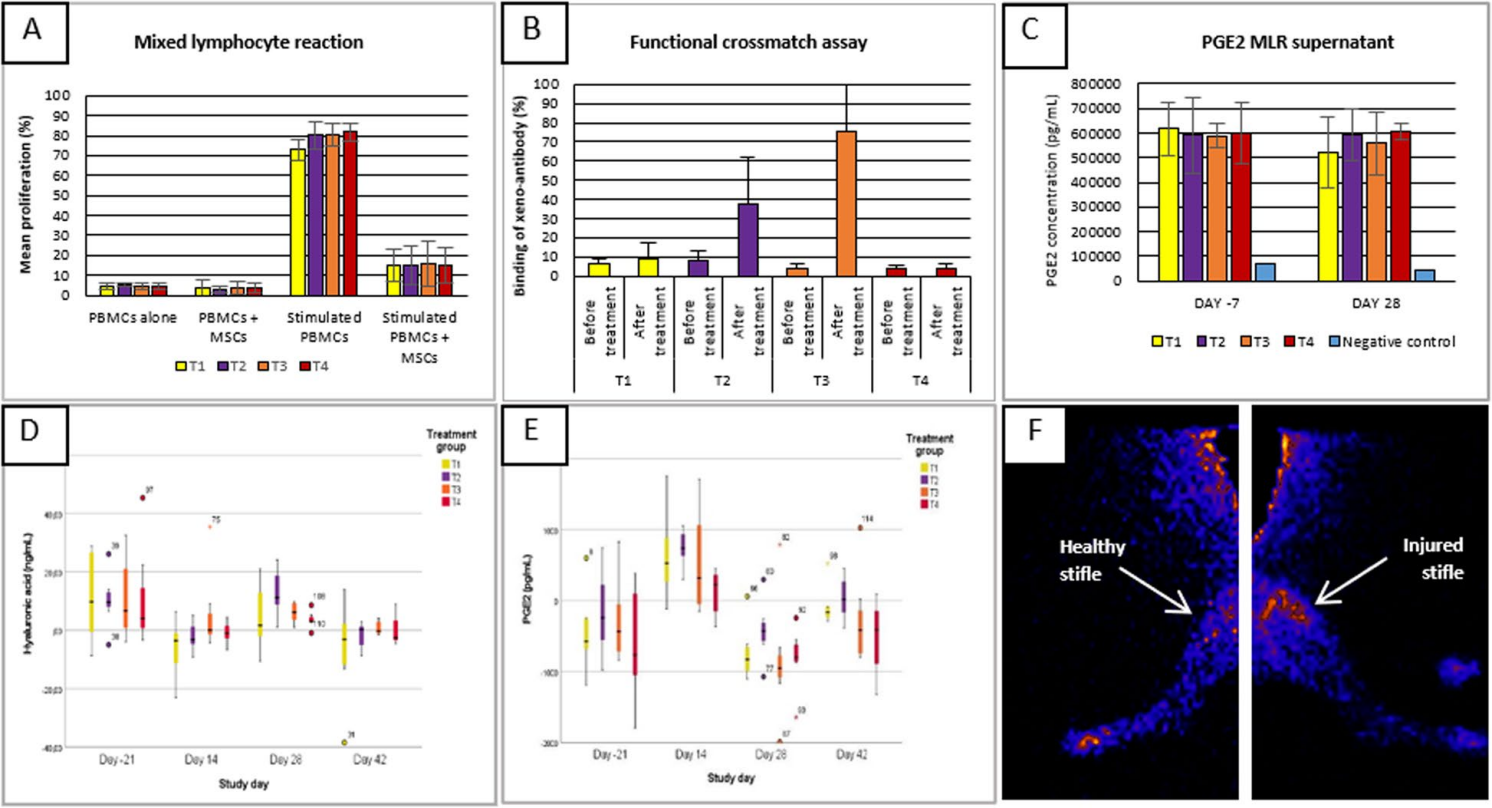
Representation of the results of the MLR assay, functional crossmatch assay, ELISA results, and biodistribution study. **A** MLR assay with negative control (PBMCs alone), positive control (stimulated PBMCs), co-culture (PBMCs + MSCs), and stimulated co-culture (stimulated PBMCs + MSCs). **B** Functional crossmatch assay evaluating humoral IgG immune response. **C** PGE2 concentration in MLR supernatants. **D** Hyaluronic acid (ng/mL) in canine serum corrected for baseline values. **E** PGE2 (ng/mL) in canine serum corrected for baseline values. **F** Biodistribution of ^99m^Tc-labeled MSCs in healthy and injured stifle. All data presented as mean ± SD. The boxplots present the 1st quartile, the 3rd quartile, and the median. In addition, outliers and extreme values are displayed

The flow cytometric crossmatch assay revealed a significant humoral immune response at day 28 in group T3 by the presence of IgG xeno-antibodies directed against the MSCs (*P* ≤ 0.001). On the other hand, in group T2, the presence of IgG xeno-antibodies was confirmed in 5 out of 8 dogs and just failed to be significant (*P* = 0.013). No xeno-antibodies were detected in the two remaining groups (T1 and T4) (T1: *P* = 0.431; T4: *P* = 0.924) (Fig. 4B). In addition, the anti-BSA ELISA confirmed the IgG xeno-antibodies were not directed against xenogeneic bovine serum proteins potentially present on the MSCs extracellular surface but rather the MSCs themselves.

### Immunomodulation assessment

#### Mixed lymphocyte reaction (MLR)

The MLR assay showed no significant difference in peripheral blood mononuclear cell (PBMC) proliferation between the negative control and the co-incubated samples (PBMCs + MSCs) over time (*P* ≥ 0.115). Furthermore, no significant difference in immunomodulation was found between day − 7 and day 28 for all IVP groups (*P* ≥ 0.172) (Fig. 4A). In addition, PBMC proliferation of the co-incubated samples was significantly lower than the positive control for all treatment groups at day − 7, day 0, and day 28 (*P* < 0.001) (Fig. 4A). Finally, PGE2 analysis of the supernatants showed significantly increased PGE2 levels in all co-culture samples (concanavalin A (ConA)-stimulated PBMCs + MSCs) (Fig. 4C).

#### ELISA analysis of serum

Four weeks post-treatment, significantly higher hyaluronic acid (HA) levels were found in the serum of group T2 compared to group T4 (*P* = 0.014). No significant changes in HA levels were found in other IVP groups or at other time points compared to group T4 (Fig. 4D). Furthermore, 2 weeks post-treatment, significantly higher serum levels of PGE2 were found in group T2 compared to group T4 (*P* < 0.001). No significant changes in PGE2 were found for other IVP groups or other time points compared to group T4 (Fig. 4E). Finally, no significant changes were found between the IVP groups and group T4 at all time points for the other investigated serum markers.

A post hoc analysis showed that higher PGE2 values are positively correlated with the improvement in cartilage and synovial scores at different time points during the study (PGE2—day 14: *P* = 0.008; day 42: *P* = 0.041) (Additional file 1: Fig. S6).

#### Biodistribution study

Investigating the biodistribution of ^99m^Tc-labeled MSCs in a canine OA model in three dogs revealed a 13.0 ± 3.9-fold higher uptake in the injured stifle joint compared to the healthy joint 24 h post-injection. Ultrasonographic examination performed 4 days after the surgery indicated a mildly increased perfusion at the injured stifle joint while only a lightly increased perfusion was observed at the level of the healthy stifle joint (Fig. 4F).

During the first day following intravenous injection of the labeled ePB-MSCs into the cephalic vein of the dogs, presence was predominantly observed in the liver, lung, heart, spleen, and urinary bladder. The highest uptake was seen in the liver with a peak 6 h post-injection. An initial uptake was present in the heart and lung and this uptake progressively decreased over time. The uptake in the spleen increased slightly following injection before decreasing subsequently. Finally, a progressively increasing uptake was observed in the urinary bladder until 24 h post-injection.

## Discussion

To the authors’ knowledge, this is the first study to investigate the dose-related safety and efficacy of systemically administered xenogeneic MSCs in dogs for the treatment of OA using a combination well described canine models. In this randomized, blinded, placebo-controlled model study, a comprehensive assessment of subjective and objective parameters evaluated the safety and efficacy of MSCs in the treatment of canine OA.

To date, the use of autologous and allogeneic MSCs has shown a positive safety profile for both humans [14, 44] and other species [17, 20, 45–47]. However, safety evaluations of xenogeneic MSCs are limited and restricted to IA injections [19, 20, 48]. In the current study, the safety of MSCs was demonstrated with no abnormal findings during clinical and pathological evaluations using IV-injected xenogeneic MSCs to dogs with OA. Next to clinical safety, the absence of a cellular immune response was confirmed for all groups using the in vitro modified MLR assay. Another MLR assay indicated the preservation of the immunomodulatory properties of the MSCs towards stimulated canine PBMCs derived from dogs which were treatment with MSCs (T1-T2-T3). However, as previously described in horses, exposure to xenogeneic or major histocompatibility complex (MHC)-mismatched allogeneic MSCs could induce a humoral immune response [48–50]. Despite MSCs being immunoprivileged because of the absence of MHCII on their cell surface, a dose-related humoral immune response (IgG antibodies) was found 2 weeks post-treatment in some dogs of group T2 and all dogs of T3. Nevertheless, the presence of xeno-antibodies directed against MSCs did not result in any clinical abnormalities and therefore does not appear to pose a safety concern. Future research is necessary to identify the surface protein on the MSCs that is responsible for the humoral immune response.

All clinical efficacy parameters including lameness, joint, animal-caretaker assessments, and objective pressure plate analysis improved significantly at 6 weeks post-treatment for each IVP group (T1 to T3) compared to the control group (T4). Furthermore, the appearance of articular heat, pain, and joint effusion was less pronounced in all three IVP groups compared to group T4. In addition, the dogs that received 0.3 × 10^6^ MSCs (group T2) showed superior results for all these clinical parameters, indicating an optimal dose effect. These results confirm that a single IV injection of xenogeneic MSCs provides a high and comparable efficacy to previous reports in feasibility, pilot, and efficacy studies using IA injection of allogeneic and xenogeneic MSCs in dogs with OA [12, 19, 20, 51–53]. This is in contrast to other animal studies, reporting that IV administration of allogeneic MSCs leads to less satisfying results compared to intra-articular administration in the treatment of naturally occurring OA [17, 20, 52, 54–57]. Furthermore, the results of the gross pathology support the clinical findings demonstrating a significant improvement in synovial, cartilage, and meniscus scoring for all treatment groups compared to the control group, indicating a positive effect on all joint structures. Moreover, the lowest synovial and cartilage scores were observed in group T2, in addition to the absence of secondary lesions on both menisci, supporting the optimal dose effect. Gross pathology results indicate that systemic MSC treatment provides a local anti-inflammatory effect which results in a protective effect on joint tissues by slowing down the degenerative processes, which can be linked to a significant improvement of all measured clinical parameters. The joint tissue protective effect of autologous and allogeneic MSCs has been described after IA injection in several canine OA model studies [58–61]. However, the current study is the first to describe this protective effect for IV-administered xenogeneic MSCs.

In contrast to the gross pathology results, no significant changes were found in histopathology between the groups. However, during the gross pathology, the complete surface of the joint and the synovium was investigated and scored, while during histopathology, only one sample per femur condyle was investigated. Sampling had to be performed adjacent to the cartilage defect as collecting a sample of cartilage directly in the created cartilage defect was not feasible. In addition, no longitudinal sections on multiple areas of the joint, cartilage, or bone samples were taken. Therefore, the histopathologic analysis had limited sensitivity compared to the gross pathology.

Due to the reported mode of action of mesenchymal stem cells and the limited relevance of pharmacokinetics and pharmacodynamics due to the absence of a pharmacological effect, dose determination studies are generally not done for MSCs. To the author’s knowledge, this is the first study to show a dose-related efficacy of MSC treatment in a canine OA model with superior efficacy using a systemic injection of 0.3 × 10^6^ MSCs and supports the hypothesized lack of linear dose-relationship for a systemic MSC administration. Compared to previously described OA studies in dogs and humans, this is a significantly lower dose [13, 17, 19, 20, 62]. However, most of the reported studies administered MSCs intra-articular into the affected joint. The systemic administration of MSCs might contribute to an increased efficacy due to its higher interaction with the immune system [17, 39]. On the other hand, the xenogeneic nature of the treatment in dogs, causing a dose-related humoral immune response, could potentially also explain the lower efficacy in group T3 receiving the highest dose. Nevertheless, as described above, the presence of xeno-antibodies did not cause any clinical side effects and did not impede a clinical effect. In addition, Olson et al. administered a higher dose of 2 × 10^6^ allogeneic MSCs/kg body weight IV in dogs and found only a significant improvement in a client-specific scoring system, but not for other tested parameters, suggesting a higher dose of MSCs might be less effective in the treatment of OA. However, this could also be due to the different sources and/or lower quality of the MSCs used in this study [17]. This dose-efficacy relationship was supported by the serum analysis showing significantly increased levels of HA and PGE2 in group T2 compared to group T4. The increased serum levels of HA might indicate a reduced cartilage degradation in treatment group T2 [63]. Furthermore, the serum results support the previously described involvement of PGE2 in the immunomodulatory mode of action of MSCs in vivo [64] which is also confirmed in the supernatants of the MLR assay. All these results imply that the systemic administration of xenogeneic MSCs initiates an optimal treatment effect in dogs with OA at a dose of 0.3 × 10^6^ cells. Furthermore, a post hoc analysis showed that the improvement of synovial and cartilage scoring is characterized by increased PGE2 serum levels.

In contrast to a local, intra-articular MSC injection, the systemic administration of MSCs could cause a reduction of OA symptoms in multiple joints, which is of high clinical relevance. This property has been assigned to the homing potential of MSCs as well as the systemic effects on the immune system. The current biodistribution study is the first to confirm the homing of xenogeneic MSCs to an OA lesion after IV administration, with a 13.0 ± 3.9-fold increased uptake in the affected joint compared to a healthy joint. In addition, this homing behavior of MSCs might be linked to an improved clinical outcome of the dogs seen in the dose-response study and therefore provide important insights into the mode of action of IV-administered xenogeneic MSCs in the treatment of canine OA. In contrast to the dose-response study, the dogs used for the biodistribution study only had a transection of the anterior cruciate ligament, without the meniscal release and creation of a bilateral cartilage defect for ethical reasons. This way, the surgical OA model could be reversed after the biodistribution study by surgery and the dogs could be adopted. Nevertheless, this difference in the model should not have an impact on the biodistribution pattern of the MSCs.

In this study, a combination of well-described canine OA models for naturally occurring OA was used to induce an accelerated model of natural OA in dogs. An OA model was chosen with the objective of creating a homogeneous study population with comparable conditions in the target joint, which allowed for a controlled comparison across groups for gross pathology, histopathology, and immunohistochemistry. However, a limitation of using a canine OA model is the time-dependent improvement of clinical parameters without treatment, which was confirmed in the current study. Even though the combination of the models has not been described previously, clinical improvement without treatment has been reported during the acute phase after induction of OA in both models separately. A progressive improvement in ground reaction forces (GRF) for both the anterior cruciate ligament transection (ACLT) and the groove model 12 weeks post-surgery in dogs has been reported [65]. Similar findings were reported by Smith et al., who described a slow increase of peak vertical ground reaction force over a period of 32 weeks after induction of the ACLT model in their placebo group [66]. Furthermore, Moreau et al. described a mild improvement in pain sensation and activity of the dogs included in a control group 8 weeks after the induction of the ACLT model [66]. Finally, a trend towards decreased lameness scores was seen in ACLT dogs during a 12-week study by Kuroki et al. [66, 67]. Nevertheless, in the current study, the dogs from all IVP groups had significantly superior and faster reduction of lameness, articular pain, and joint effusion scores compared to the negative control group. Future studies are needed to evaluate and confirm the effect of MSCs in naturally occurring canine OA.

Another limitation of this study is the unsuccessful collection of synovial fluid due to the small size of the joints of the dogs. Therefore, analysis of the synovial fluid could not be performed in this study. A technical limitation of this study was the sampling technique used for the histopathologic analysis, indeed only one sample per tissue was taken which reduced the significance of this evaluation.

## Conclusions

In conclusion, this model-based study is the first to confirm the efficacy and safety of systemically administered xenogeneic MSCs in dogs with OA. Furthermore, significant dose-related properties were seen in terms of immunomodulation and joint tissue protection, supporting their potential to slow down disease progression. Furthermore, the cells show homing to the lesion site. These results indicate that in the future, xenogeneic MSCs could potentially be used as a widely accessible treatment to address the clinical signs as well as pathological changes of OA across different species, including humans. However, future studies are needed to confirm the mode of action of the xenogeneic MSCs and their efficacy and safety in dogs with naturally occurring OA.

### Supplementary Information

**Additional file 1:** **Supplementary information.**

## Data Availability

The datasets used and/or analyzed during the current study are available from the corresponding author upon reasonable request.

## Abbreviations

ACTL: Anterior cruciate ligament transection
CBPI: Canine Brief Pain Inventory
C3a: Complement factor C3a
CFSE: Carboxyfluorescein succinimidyl ester
COMP: Cartilage oligomeric matrix protein
ConA: Concanavalin A
C2C: Collagen type II cleavage
DMEM: Dulbecco’s modified Eagle medium
DMSO: Dimethylsulfoxide
EDTA: Ethylenediaminetetraacetic acid
ePB-MSC: Equine peripheral blood-derived mesenchymal stem cell
HA: Hyaluronic acid
HBSS: Hank’s Balanced Salt Solution
IA: Intra-articular
IFN-γ: Interferon-gamma
IV: Intravenous
IVP: Investigational veterinary product
LFC: Lateral femoral condyle
MF: Mean force
MFC: Medial femoral condyle
MLR: Mixed lymphocyte reaction
MMF: Mean maximal force
MHC: Major histocompatibility complex
MSC: Mesenchymal stem cell
NSAID: Non-steroidal anti-inflammatory drug
OA: Osteoarthritis
OARSI: Osteoarthritis Research Society International
PBMC: Peripheral blood mononuclear cell
PGE2: Prostaglandin E2
PIS: Pain interference score
PSS: Pain severity score
QOL: Quality of life
ROI: Regions of interest
ROM: Range of motion
SI: Symmetry index
TGF-β1: Transforming growth factor beta 1
^99m^Tc: ^99m^Technetium
vWF: von Willebrand factor
