# Supplementary material for: Low-dose xenogeneic mesenchymal stem cells target canine osteoarthritis through systemic immunomodulation and homing
Source: Arthritis Res Ther. 2023 Oct 3;25:190. doi: 10.1186/s13075-023-03168-7 (PMC10546732; doi:10.1186/s13075-023-03168-7)
Supplement: Supplementary file 1 — Additional file 1: Supplementary information. [file 13075_2023_3168_MOESM1_ESM.docx]

**Assessments by Pathologist: Definitions of scores and evaluation criteria**

Post-mortem examination consisting of gross pathology, histopathology and immunohistochemistry according to OARSI recommendations (Cook et al., 2010) was performed as described below.

1. **Gross pathology**

The assessment by gross pathology was performed based on the parameters listed below.

- 1. **Synovial pathology**

The affected joint was assessed for synovial pathology and scored as defined below based on the worst pathology noted in the tissue below the patella and the medial and lateral sides of the joint capsule from patellar ligament to lateral and medial attachments.

**Supplementary Table 1: Synovial pathology scoring**

| **Score** | **Definition** |
| --- | --- |
| **0** | Normal = opal white, semitranslucent, smooth, with sparse well defined blood vessels |
| **1** | Slight =focal involvement, slight discoloration, visible proliferation/fimbriation/thickening, notable increase in vascularity |
| **2** | Mild = diffuse involvement, slight discoloration, visible proliferation/fimbriation/thickening, notable increase in vascularity |
| **3** | Moderate = diffuse involvement, severe discoloration, consistent notable proliferation/ fimbriation/thickening, moderate vascularity |
| **4** | Marked = diffuse involvement, severe discoloration, consistent and marked proliferation/ fimbriation/thickening, diffuse hypervascularity |
| **5** | Severe = diffuse involvement, severe discoloration, consistent and severe proliferation /fimbriation/thickening, thickening to the point of fibrosis, and severe hypervascularity |

- 1. **Cartilage scoring of weight bearing surfaces**

The affected joint was assessed for cartilage scoring of the weight bearing surfaces MFC (medial femoral condyle) and LFC (lateral femoral condyle), as defined below. One overall cartilage score was assigned based on the most severe pathology noted.

**Supplementary Table 2: Cartilage scoring**

| **Score** | **Definition** |
| --- | --- |
| **0** | Smooth surface |
| **1** | Slightly fibrillated/roughened surface |
| **2** | Fibrillated surface with focal partial thickness lesions |
| **3** | Deep lesions with surrounding damage |
| **4** | Large areas of severe damage |

- 1. **Score medial and lateral menisci**

The affected joint was assessed for scoring of each zone (anterior (cranial), middle, and posterior (caudal) thirds) of each the medial and lateral meniscus as defined below.

**Supplementary Table 3: Meniscus scoring**

| **Zone of meniscus** | **Anterior** | **Middle** | **Posterior** | **Definition** |
| --- | --- | --- | --- | --- |
| **Score** | 0 | 0 | 0 | None |
|  | 1 | 1 | 1 | Fibrillation only |
|  | 2 | 2 | 2 | Incomplete tear or tears |
|  | 3 | 3 | 3 | Complete tear or tears |
|  | 4 | 4 | 4 | Complete disruption of structure (maceration of tissue) |

The scores of each meniscus zone were totaled to obtain the total score for each meniscus with a maximum score of 12.

- 1. **Evaluation ectopic tissue at injection site (skin+vessel)**

The skin and the blood vessel at the injection site will be assessed for the presence of ectopic tissue and scored as follows:

**Supplementary Table 4: Presence of ectopic tissue**

| **Score** | **Presence of ectopic tissue** |
| --- | --- |
| **0** | Absent |
| **1** | Present |

1. **Histopathology**

The assessment by histopathology was performed based on the parameters listed below using hematoxylin-eosin stain. When a section included several local areas of pathology or local and multi-focal pathology, then the scores were added to derive a total score for the section. The score was based on the most severe pathology seen in each area of the section that was evaluated.

- 1. **Histopathology of each joint surface**

One section had to be evaluated in its entirety for each of the following compartments of joint surface, i.e. medial femoral condyle (MFC) and lateral femoral condyle (LFC).

Tables 5 and 6 provide the scoring systems that were used for the relevant category of cartilage and chondrocyte pathology.

**Supplementary Table 5: Cartilage structure**

| **Severity of pathology**  **Characteristics** | | **Area of section affected** | | | |
| --- | --- | --- | --- | --- | --- |
|  |  | **None** | **Local (approx 1/3)** | **Multi-focal (approx 2/3)** | **Global (>2/3)** |
| A | Normal volume, smooth surface with all zones intact | 0 | 0 | 0 | 0 |
| B | Surface undulations including fissures in surface/upper zone and/or pannus tissue formation on surface | 0 | 1 | 2 | 3 |
| C | Fissures to mid zone and/or erosion of surface/upper zone | 0 | 2 | 4 | 6 |
| D | Fissures that extend to deep zone and/or erosion through mid zone | 0 | 3 | 6 | 9 |
| E | Full thickness loss of cartilage | 0 | 4 | 8 | 12 |

**Supplementary Table 6: Chondrocyte pathology**

| **Severity of pathology**  **Characteristics** | | **Area of section affected** | | | |
| --- | --- | --- | --- | --- | --- |
|  |  | **None** | **Local (approx 1/3)** | **Multi-focal (approx 2/3)** | **Global (>2/3)** |
| A | Normal | 0 | 0 | 0 | 0 |
| B | Loss of cells in the surface zone or relative increased density with occasional superficial clusters | 0 | 1 | 2 | 3 |
| C | Small cell clusters (2-4 cells/cluster)* predominate | 0 | 2 | 4 | 6 |
| D | Large cell clusters (≥5 cells/cluster) predominate | 0 | 3 | 6 | 9 |
| E | Cell loss (necrosis/apoptosis) predominates | 0 | 4 | 8 | 12 |

* in any cartilage zone

- 1. **Histopathology of Synovium**

For the synovial assessment one section of tissue had to be evaluated from axial compartment of the affected joint. The section had to be representative of the entire tissue. Supplementary Table **7** provides the scoring system that was used for the synovial assessment.

**Supplementary Table 7: Cell infiltration of synovium**

| **Severity of pathology**  **Characteristics** | | **Area of section affected** | | | |
| --- | --- | --- | --- | --- | --- |
|  |  | **None** | **Local (approx 1/3)** | **Multi-focal (approx 2/3)** | **Global (>2/3)** |
| A | No cellular infiltration | 0 | 0 | 0 | 0 |
| B | Mild to moderate inflammatory cell infiltrates including small lymphoid follicles | 0 | 1 | 2 | 3 |
| C | Marked, diffuse inflammatory cell infiltrates including large lymphoid follicles | 0 | 2 | 4 | 6 |

- 1. **Ectopic tissue at MFC,LFC,MTP,LTP,synovium/ joint capsule, injection site**

The MFC, LFC, MTP, LTP, synovium/joint capsule, injection site wasassessed for the presence of ectopic tissue and scored as follows:

**Supplementary Table 8: Ectopic tissue**

| **Score** | **Presence of ectopic tissue** |
| --- | --- |
| **0** | Absent |
| **1** | Present |

1. **Immunohistochemistry**

The assessment by immunohistochemistry will be performed for the different components of cartilage of the following joint surfaces (i.e. MFC and LFC) and to assess the vascularity in the synovium/joint capsule based on the parameters listed below and documented on **Error! Reference source not found.**.

**Supplementary Table 9: Immunohistochemistry**

| COMP : area percentage |
| --- |
| Collagen type II: area percentage |
| Alcian blue stain for glycosaminoglycans : area percentage |
| vWF: area percentage |

**Reference**

Cook, J.L., Kuroki, K., Visco, D., Pelletier, J.P., Schulz, L., Lafeber, F.P., 2010. The OARSI histopathology initiative - recommendations for histological assessments of osteoarthritis in the dog. Osteoarthritis Cartilage Suppl 3,S66-79.

**Representative images of immunohistochemistry**


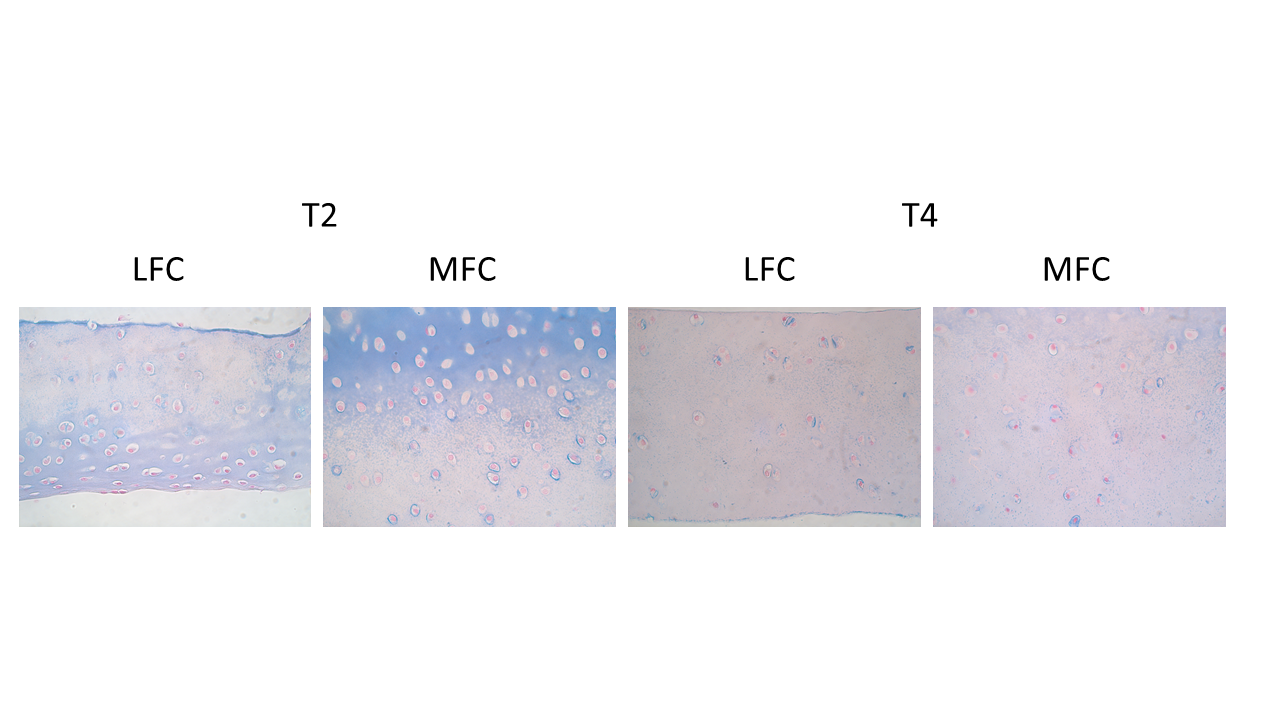


Supplementary Figure 1. Representative images of immunohistochemistry using alcian blue (AB) staining of the lateral femoral condyle (LFC) and the medial femoral condyle (MFC) of treatment group 2 (T2) and treatment group 4 (T4). The AB staining confirms the presence of glycosaminoglycans in the cartilage of both treatment groups.


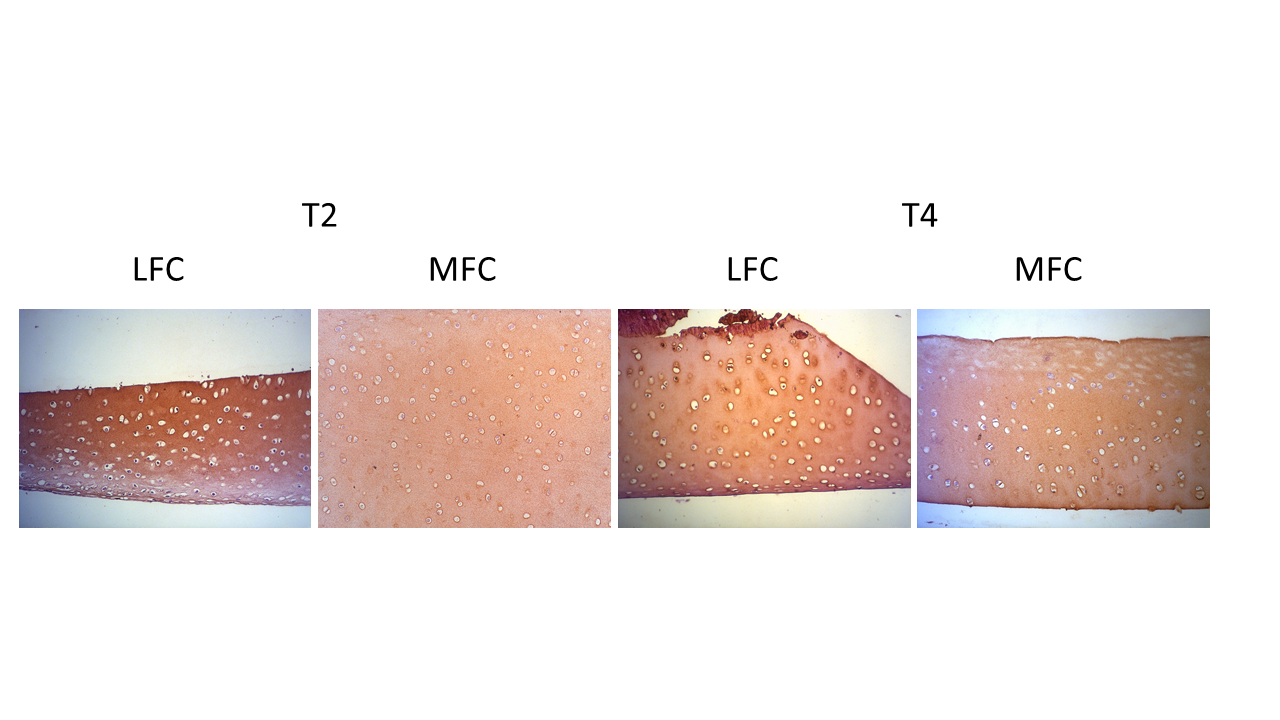


Supplementary Figure 2. Representative images of Collagen type II immunohistochemistry staining. The mean area percentage for collagen type II in the lateral femoral condyle (LFC) and the medial femoral condyle (MFC) of treatment group 2 (T2) and treatment group 4 (T4) was similar.


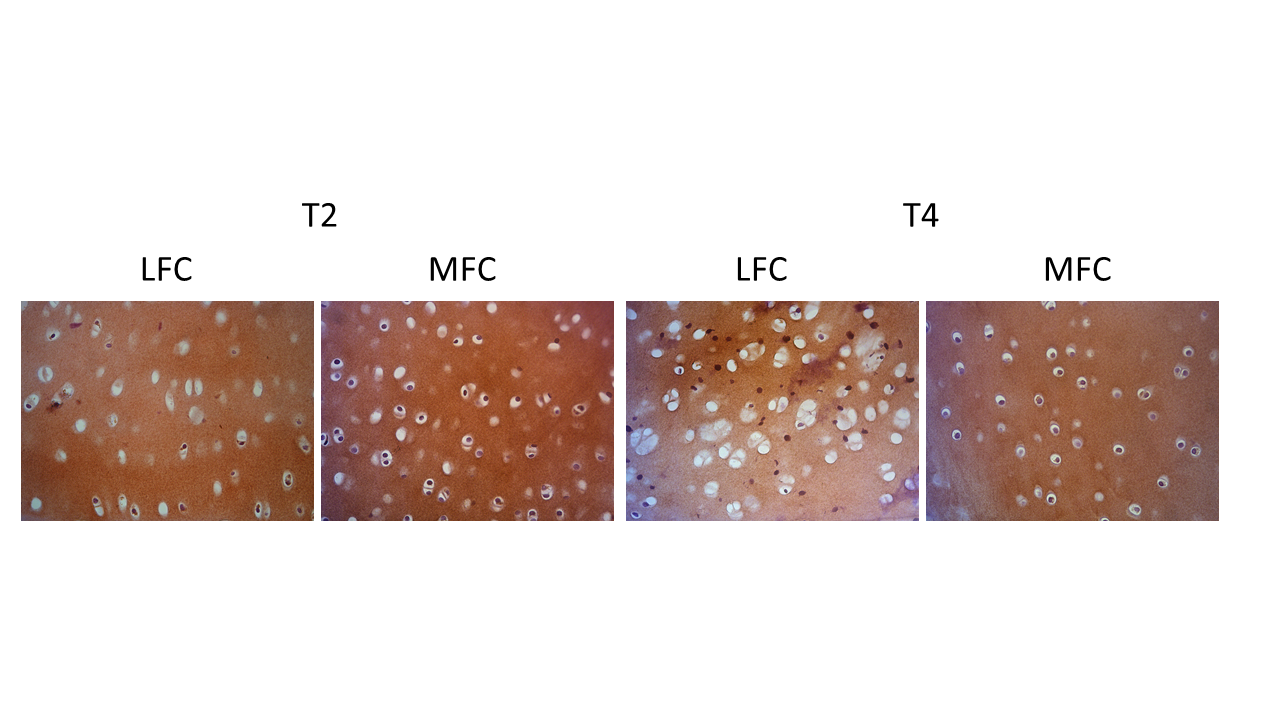


Supplementary Figure 3. Representative images of cartilage oligomeric matrix protein (COMP) immunohistochemistry staining. The mean area percentage for COMP in the lateral femoral condyle (LFC) and the medial femoral condyle (MFC) of treatment group 2 (T2) and treatment group 4 (T4) was similar.


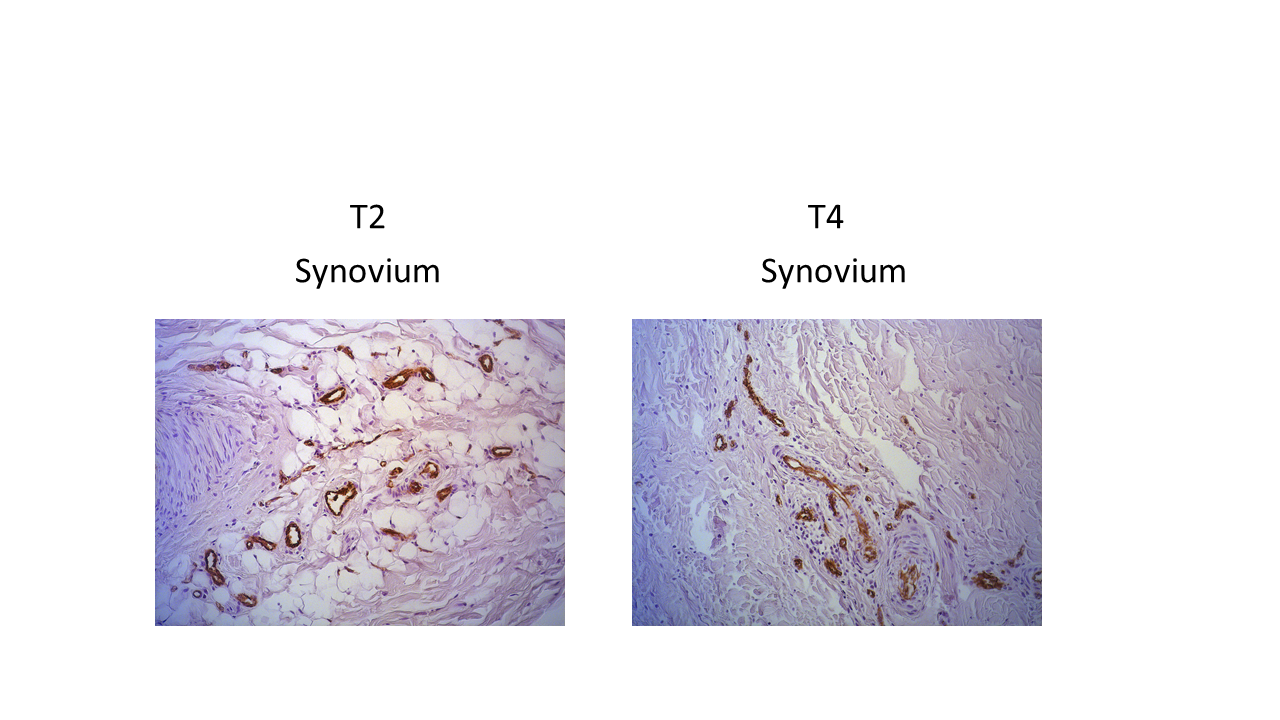


Supplementary Figure 4. Representative images for Von Willebrand factor expression demonstrating the vascularization in the synovium of treatment group 2 (T2) and treatment group 4 (T4).


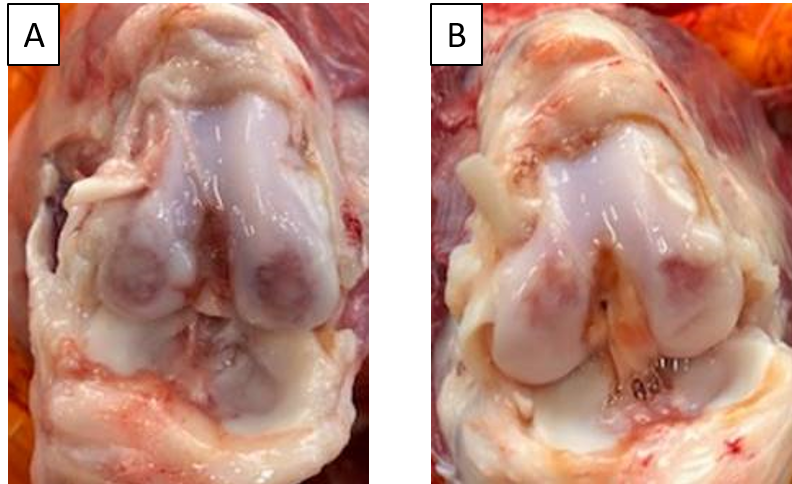


Supplementary figure 5. Example of an affected stifle joint at necropsy on day 42±3 in the placebo control group T4 (A) and the IVP group T2 (1x recommended dose) (B)


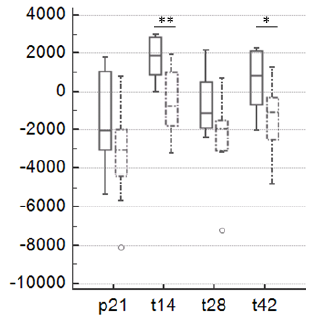


Supplementary figure 6**.** Boxplot representations of the changes in PGE2 concentrations (ng/mL) compared to day 0 from the defined controls and cases based on cartilage and synovial scores at day (p/t) -21, 14, 28 and 42.

Full bars: controls, dashed bars: cases. (*:p<0.05, **:p<0.01).
